# Synthesis of Biochar-Supported K-doped g-C_3_N_4_ Photocatalyst for Enhancing the Polycyclic Aromatic Hydrocarbon Degradation Activity

**DOI:** 10.3390/ijerph17062065

**Published:** 2020-03-20

**Authors:** Fayun Li, Meixia Lin

**Affiliations:** 1 School of Ecological Technology and Engineering, Shanghai Institute of Technology, Shanghai 210418, China; 2 National & Local United Engineering Laboratory of Petroleum Chemical Process Operation, Optimization and Energy Conservation Technology, Liaoning Shihua University, Fushun 113001, China; 3 College of Resources and Environment, Hunan Agricultural University, Changsha 410128, China; linmx2020@163.com

**Keywords:** biochar, graphite phase carbon nitride, photocatalytic degradation, naphthalene

## Abstract

The development of novel and green photocatalysts have attracted considerable attentions due to their excellent performance for environmental remediation, especially for the degradation of persistent pollutants. In this work, the biochar-supported K-doped g-C_3_N_4_ composites with the high photocatalytic activity under visible light irradiation was prepared by the calcination-impregnation method. The crystal structure, apparent morphology and functional group composition of the as-prepared photocatalytic materials were studied by X-ray diffraction (XRD), scanning electron microscopy (SEM) and Fourier transform infrared spectroscope (FTIR). Moreover, the characterization of UV-Vis diffuse reflectance spectra (UV-Vis DRS) and photoluminescence technique (PL) verified the good optical properties of resultant samples. Naphthalene was selected as the representative compound to evaluate the photocatalytic performance of the prepared photocatalysts under visible light irradiation. The evaluation results showed that the biochar-supported K-doped g-C_3_N_4_ composites exhibited excellent photocatalytic activity (82.19%). Moreover, the photocatalytic degradation rate basically remained unchanged after five cycles, indicating the good stability of the prepared photocatalysts. In addition, a possible mechanism for the photodegradation process was proposed on the basis of the main intermediates detected by gas chromatography-mass spectrometer (GC-MS). This study may provide a promising approach for the polycyclic aromatic hydrocarbon degradation by waste utilization of agricultural biomass and increasing the photocatalytic performance of pure g-C_3_N_4_.

## 1. Introduction

The pollution of petroleum hydrocarbons is one of the environmental problems in the petrochemical industry in recent years [1]. Petroleum hydrocarbons are complex mixtures composed of alkanes, olefins and polycyclic aromatic hydrocarbons (PAHs). Petroleum hydrocarbon leakage may cause serious and persistent water and soil pollution. For example, due to petroleum hydrocarbon contamination, the quality and visibility of water body decrease, and the fertility and water-holding capacity of soil are also reduced [2]. In particular, PAHs have the characteristics of high toxicity, mutagenicity and carcinogenicity [3]. Unfortunately, PAHs deposited in soil and groundwater pose a tremendous threat to the entire ecosystem. Naphthalene, as a representative substance of polycyclic aromatic hydrocarbons (PAHs), has been classified as a priority control pollutant by the Environmental Protection Agency of the United States and has been identified to be hazardous to the health of humans and animals [4]. Therefore, the effective treatment of naphthalene deposited in water and soil systems is extremely important for ecological environment safety. Conventional physicochemical techniques and biotechnologies have been developed to remove naphthalene from various systems, such as activated carbon adsorption [5], oxidative decomposition [6] and biological treatment [7]. However, these methods have some limitations [8]. Physical adsorption merely transfers naphthalene from one phase to another and cannot achieve complete degradation [9]. Chemical oxidation is harsh and requires strong oxidants or metal ions, so it can easily cause secondary pollution [10]. In addition, biological treatment technologies were proven to be inefficient, because they were time-consuming and difficult to be controlled [11]. Thus, it is necessary to develop advanced and environmentally friendly alternative technologies to effectively decompose naphthalene.

Photocatalytic technologies can make full use of solar energy to directly convert light energy into chemical energy, thus promoting chemical reactions. They have been successfully applied in clean energy production [12], environmental pollution remediation [13], chemical synthesis [14] and other fields. Graphitic carbon nitride (g-C_3_N_4_) is a novel metal-free visible-light-induced organic semiconductor with good chemical stability and well-suited band positions [15] and has been successfully applied in water-splitting [16], photofixation of CO_2_ [17] and pollutant degradation [18]. It can be easily prepared via thermal polymerization and electrochemical deposition with abundant and cheap precursor materials like urea, dicyandiamide, thiourea and melamine [19]. However, the disadvantages of low specific surface area, high photogenerated electron and hole pairs recombination and low visible light utilization efficiency still limit its application scope [20]. Many attempts have been made to improve the photocatalytic performance of pure g-C_3_N_4_ materials, such as doping with metal atoms [21] or nonmetal atoms [22], dye sensitization [23] and copolymerization [24].

Recently, the modifications of g-C_3_N_4_ with metal atoms have been widely concerned. Chen et al. [25] reported that Fe-doping of g-C_3_N_4_ significantly tuned its electronic structure, extended the spectral absorption range and greatly enhanced the photocatalytic activity of the system for the oxidation of benzene to phenol. Wang et al. [26] introduced K into the g-C_3_N_4_ matrix by a facile thermal polymerization with KBr as the K source. The experiments of photocatalytic hydrogen production under visible-light irradiation demonstrated that potassium-doping effectively inhibited the growth of carbon nitride grains, increased the π-conjugated systems and enhanced light-harvesting properties. Moreover, the photocatalytic hydrogen evolution rate of the K-doped g-C_3_N_4_ nanosheets was about 5.6 times of that of pure g-C_3_N_4_. Zhang et al. [27] also prepared K-doped g-C_3_N_4_ with high photocatalytic activity via thermal polymerization of dicyandiamide and KI in the atmosphere, which distinctly enhanced the degradation rate of phenol and MB compared to bulk g-C_3_N_4_. Therefore, doping g-C_3_N_4_ with metals, especially alkali metals, was reported as one of the most promising strategies for the photocatalytic degradation of organic pollutants under visible-light irradiation.

In addition, some researchers increased the amount of carbon materials, such as graphene, carbon nanosheets and carbon quantum dots, to improve the photocatalytic activity of bulk g-C_3_N_4_. Graphene, possessing excellent characteristics of a large π-conjugation structure, efficient charge transfer performance and superior photocatalytic activity, is a potential nanocarbon material for the degradation of pollutants in the environment field [28]. Du et al. [29] successfully prepared the hybrid graphene/g-C_3_N_4_ nanocomposite, which displayed an enhanced optical absorption in the visible region and a wider photocatalytic application scope compared to pure g-C_3_N_4_. Therefore, coupling g-C_3_N_4_ with carbon materials is a good choice to prepare high-performance photocatalysts. To the best of our knowledge, the biochar, as a novel and ecofriendly carbon material, possesses high conductivity, stability and photoelectric characteristics. Therefore, it is of great significance to utilize abundant and renewable agricultural waste materials, such as corn straw and rice straw, for preparing biochar and modified g-C_3_N_4_. It provides a way to utilize organic pollutants.

In this work, with dicyandiamide and corn straw powder as raw materials, we synthesized recoverable biochar-supported K-doped g-C_3_N_4_ photocatalysts by the high temperature calcination and dipping method at 550 °C. The phase structure, morphology, chemical composition and optical properties of photocatalysts were characterized with X-ray diffractometer (XRD), scanning electron microscope (SEM), Fourier transform infrared (FTIR) spectroscope, UV-visible diffuse reflectance spectroscope (DRS) and photoluminescence (PL) spectroscope. On the basis of the above analyses, the photocatalytic activity and stability for the degradation of naphthalene were investigated under visible light irradiation. The possible mechanism of naphthalene degradation was also discussed based on the intermediates determined with gas chromatography-mass spectrometer (GC-MS).

## 2. Materials and Methods

### 2.1. Synthesis of Photocatalysts

Dicyandiamide, potassium hydroxide, isopropanol, ascorbic acid, ethylenediamine tetraacetic acid and naphthalene were purchased from Sinopharm Chemical Reagent Co., Ltd., Shenyang, China and directly used without further purification.

Biochar (corn stalk biomass waste), pure g-C_3_N_4_ and K-doped g-C_3_N_4_ were prepared according to previous methods [27,30,31] with slight modifications. Potassium-doped g-C_3_N_4_ was synthesized with the calcination method according to the doping ratio of 1.40%. Biochar-supported K-doped g-C_3_N_4_ photocatalyst was fabricated with the calcination-impregnation method. The typical preparation procedure of biochar-supported K-doped g-C_3_N_4_ photocatalysts was described as follows. A certain proportion of corn straw biochar and potassium-doped g-C_3_N_4_ powder was dissolved in ultrapure water to obtain a suspension, which was dispersed uniformly by ultrasonic vibration, stirred overnight on a magnetic stirrer, evaporated to dryness in a water bath and then dried in an oven for a period. The dried materials were calcined at a high temperature again, cooled to room temperature and fully ground to obtain biochar-supported K-doped g-C_3_N_4_ composite photocatalysts (the mass ratios of biochar to K-doped g-C_3_N_4_ were respectively 1:0.8, 1:1 and 1:1.2).

### 2.2. Characterization of Photocatalysts

The crystallinity of the prepared samples was recorded by an X-ray diffractometer (XRD) on a Rigaku D/max-2400 instrument with Cu-Kα radiation. The morphologies and structures of the samples were collected by using a scanning electron microscope (S-4200, Hitachi, Tokyo, Japan) with an accelerating voltage of 15 kV. Chemical-bonding status was assessed by Fourier transform infrared (FT-IR) spectra (Nicolet 6700 IR spectrometer, Thermo Fisher Scientific, Waltham, MA, USA) in the range of 400 to 4000 cm^−1^. UV-Vis diffuse reflectance spectral measurement was carried out on a JASCO V-550 model UV-Vis spectrophotometer (JASCO, Tokyo, Japan) to determine the optical band gap of the photocatalysts. Photoluminescence (PL) spectra were measured at room temperature with a fluorospectrophotometer (FP-6300, JASCO, Tokyo, Japan) using a Xe lamp as an excitation source under the excitation wavelength of 380 nm.

### 2.3. Photocatalytic Experiments

Naphthalene was selected as the representative substrate to evaluate the photocatalytic performance of prepared photocatalysts under visible light irradiation. Considering that the prepared photocatalyst has a certain adsorption potential, we adopted two protocols to evaluate its photocatalytic activity. In the first protocol, prior to illumination, 50 mg of photocatalysts were dispersed in an aqueous solution of naphthalene (100 mL and 20 mg L^−1^), and the suspension was stirred for 30 min in the dark to achieve the absorption-desorption equilibrium. For the photocatalytic experiments, the mixture was exposed to a 200-W high-pressure sodium lamp with the main emission in the range of 400~800 nm (the UV light portion of the sodium lamp was filtered by 0.5-mol L^−1^ NaNO_2_ solution) [32]. The experimental temperature was maintained at 30 °C with water circulation, and air was bubbled through the solution at a flow rate of 80 mL min^−1^. According to certain time intervals, 5 mL of the suspension was taken with a syringe and immediately centrifuged to remove the particles. The concentrations of naphthalene were measured by UV-2450 spectrophotometer (Shimadzu, Kyoto, Japan) at a wavelength of 221 nm. In the second protocol, the suspension was directly exposed to visible light for adsorption and photo-degradation. The active species-trapping experiments were performed according to the same procedure to the photocatalytic experiments, except that additional isopropanol, ascorbic acid and ethylenediamine tetraacetic acid were added during the photocatalytic degradation process.

The stability of the photocatalysts were investigated by recycling experiments. The photocatalytic degradation of the recycled photocatalysts was carried out under visible light irradiation. After each cycle, the photocatalysts were collected through filtration, washed and dried before reuse.

### 2.4. Determination of Intermediates

The degradation intermediates of naphthalene were analyzed with gas chromatography (GC) coupled with a mass spectrometer (MS) technique (Agilent 7890A/5975C, Agilent, Santa Clara, CA, USA). The gas chromatograph was fitted with an HP-5MS column (30 m × 0.25 mm × 0.25 μm). The column temperature was linearly programmed to increase from 35 to 300 °C at a rate of 5 °C min^−1^. The mass spectrometric data were acquired at 70 eV in electron ionization mode. Afterwards, the possible degradation pathway of naphthalene was proposed according to the intermediates.

## 3. Results and Discussion

### 3.1. Characterization Results of Samples

#### 3.1.1. XRD Patterns

**Figure 1:**
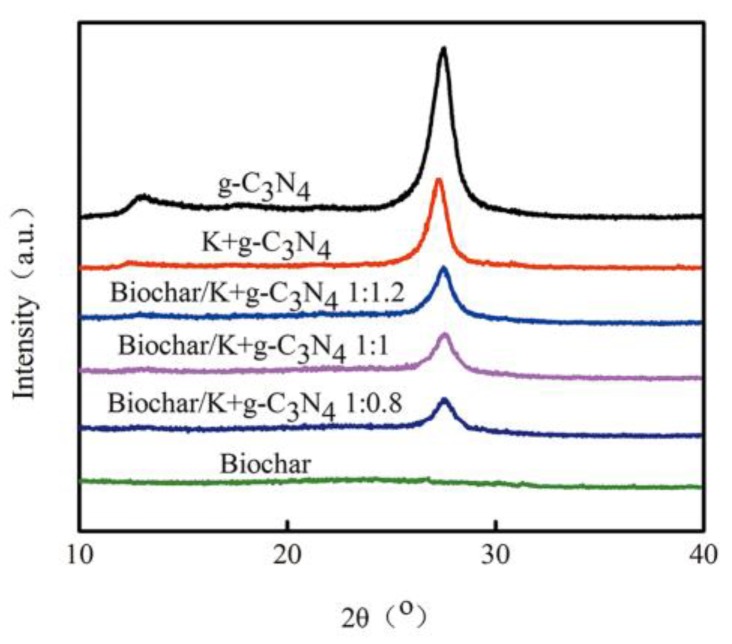
X-ray diffraction (XRD) patterns of biochar, g-C_3_N_4_, K-doped g-C_3_N_4_ and biochar-supported K-doped g-C_3_N_4_.

The XRD patterns of biochar, g-C_3_N_4_, K-doped g-C_3_N_4_ and biochar-supported K-doped g-C_3_N_4_ are shown in Figure 1. The corn straw biochar was amorphous carbon, and its XRD pattern did not show characteristic peaks. A weaker diffraction peak at 13.1° and a stronger one at 27.5° were observed in the XRD pattern of g-C_3_N_4_. The peak at 13.1° was ascribed to an in-plane structural repeating motif of tri-s-triazine units and indexed as the (100) peak with the *d* value of 0.675 nm. The peak at 27.5° was assigned to interlayer stacking of the conjugated aromatic segments with a distance of 0.332 nm and indexed as the (002) peak of the stacked aromatic system [33]. However, the diffraction peak intensity of K-doped g-C_3_N_4_ was largely weakened, indicating that the K incorporation resulted in the decrease in the crystallinity of g-C_3_N_4_, because the increased dopants inhibited crystal growth. Moreover, its diffraction peak positions showed a downshift toward a lower 2θ value, indicating the shorter-range order and larger distance of the interlayer stacking of aromatic segments [34]. In the XRD pattern of the biochar-supported K-doped g-C_3_N_4_, the diffraction peaks at 13.1° almost disappeared, indicating that the inner structure of carbon nitride was destroyed to a certain degree.

#### 3.1.2. SEM Images

**Figure 2:**
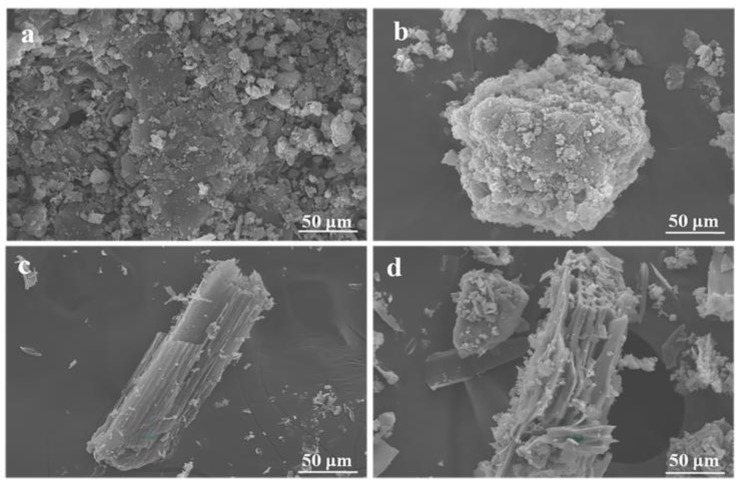
Scanning electron microscopy (SEM) images of (**a**) g-C_3_N_4_, (**b**) K-doped g-C_3_N_4_, (**c**) biochar and (**d**) biochar-supported K-doped g-C_3_N_4_.

The morphologies of biochar and photocatalyst powder were examined with SEM analysis (Figure 2). Similar to the analogue graphite, g-C_3_N_4_ displayed a large particle shape with the layered structure (Figure 2a). K-doped g-C_3_N_4_ exhibited much smaller polymer particles than that of g-C_3_N_4_, demonstrating that the structure of the catalyst particles was changed to some degree by the dopant atoms with large diameters, as confirmed by XRD results. Figure 2c shows the morphology of the biochar synthesized by calcination at 550 °C, and its main body is a keel structure with a large number of pores on the top. The K-doped g-C_3_N_4_ particles were dispersed nonuniformly on the surface of the biochar, and no apparent agglomeration was observed. A large contact area between the photocatalyst and organic pollutant largely increased the reactive sites and facilitated the photocatalytic activity.

#### 3.1.3. FT-IR Spectra

**Figure 3:**
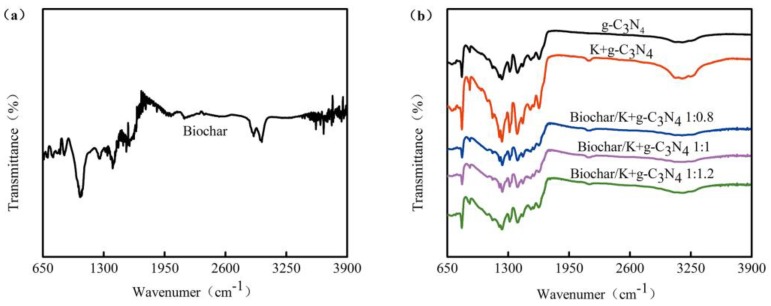
Fourier transform infrared (FTIR) spectra of (**a**) biochar, (**b**) g-C_3_N_4_, K-doped g-C_3_N_4_ and biochar-supported K- doped g-C_3_N_4_.

FTIR spectra of as-prepared biochar, g-C_3_N_4_, K-doped g-C_3_N_4_ and biochar-supported K-doped g-C_3_N_4_ are shown in Figure 3. In the spectra of the biochar, the peak at 3600 cm^−1^ is attributed to the -OH stretching of the cellulosic component in the biochar; the peaks at 2990 cm^−1^ and 2894 cm^−1^ are attributed to -CH_2_- symmetric stretching of an alkyl moiety on the biochar surface; the absorption peaks at 1400 cm^−1^ and 1054 cm^−1^ correspond to C=O and C-O-C stretching vibrations of a phenolic moiety and polysaccharides components of the biochar, respectively [35]. The broad absorption peak of g-C_3_N_4_ in the region of 3102~3373 cm^−1^ corresponds to the N-H stretching of uncondensed amine groups [36]; a series of peaks in the region of 1016~1727 cm^−1^ are attributed to the C-N and C=N vibrations of aromatic CN heterocycles; the peak at 806 cm^−1^ can be assigned to the particular bending vibration mode of the s-triazine rings of g-C_3_N_4_; the bands at 889 cm^−1^ can be assigned to the bending mode of the heptazine unit and terminal amine groups [37]. Furthermore, the spectra of K-doped g-C_3_N_4_ exhibits the same bands as pure g-C_3_N_4_, except a band at 2200 cm^−1^ originated from the defects of a cyanogen group stretch caused by the incomplete polymerization or amino groups dropped off from the surface of g-C_3_N_4_ [38]. The FTIR spectra of biochar-supported K-doped g-C_3_N_4_ are similar to those of g-C_3_N_4_, indicating that the biochar did not destruct the chemical structure of g-C_3_N_4_, but abundant functional groups on the surface of the biochar effectively promoted the dispersion of the composite photocatalysts in polar solvents. 

#### 3.1.4. UV-vis Diffuse Reflectance Spectra

**Figure 4:**
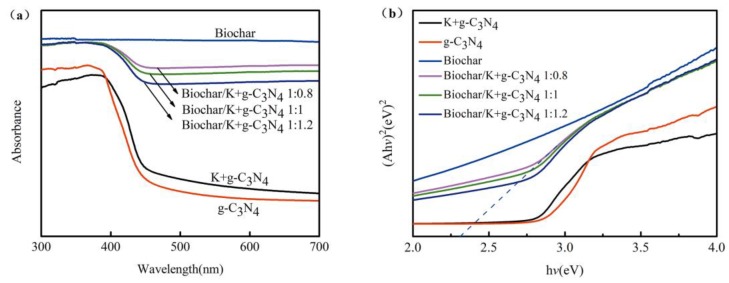
(**a**) UV-visible diffuse reflectance spectroscope (DRS) and (**b**) Tauc plots of biochar, g-C_3_N_4_, K-doped g-C_3_N_4_ and biochar-supported K-doped g-C_3_N_4_.

UV-vis diffuse reflectance spectra of the prepared samples are shown in Figure 4. The intersection point of the tangent of the curves and the abscissa are used to estimate the absorption edge. The biochar shows the strongest adsorption to the light of all wavelengths. Pure g-C_3_N_4_ exhibits an absorption edge around 460 nm, corresponding to a band gap of 2.69 eV [39]. The intrinsic absorption edge of K-doped g-C_3_N_4_ is red-shifted compared with that of pure g-C_3_N_4_, confirming that K-doping could modulate its electronic structure, reduce the band gap and enhance the absorption of visible light [40]. In the spectra of biochar-supported K-doped g-C_3_N_4_, the absorption edges show the obvious red shifts, and the absorption intensity remarkably increases, suggesting that the introduction of amorphous carbon sources effectively promoted the separation of photogenerated electron-hole pairs and enhanced their response range to visible light. In Figure 4b, compared with pure g-C_3_N_4_, the energy gap of biochar-supported K-doped g-C_3_N_4_ is significantly narrowed; the narrower energy gap of biochar-supported K-doped g-C_3_N_4_ is able to make full use of the visible light, further resulting in a higher photocatalytic performance.

#### 3.1.5. PL Spectra

**Figure 5:**
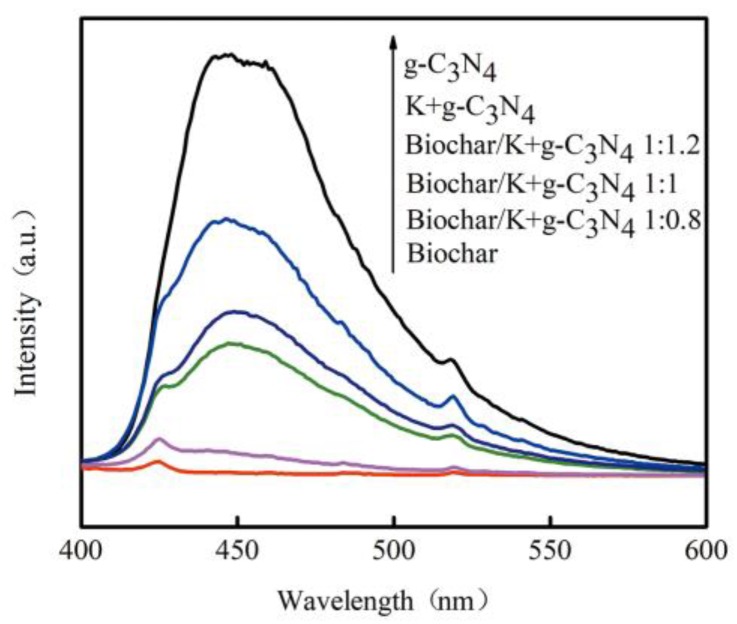
Photoluminescence (PL) spectra of biochar, g-C_3_N_4_, K-doped g-C_3_N_4_ and biochar-supported K-doped g-C_3_N_4_.

Figure 5 illustrates the PL emission spectra of biochar, g-C_3_N_4_, K-doped g-C_3_N_4_ and biochar-supported K-doped g-C_3_N_4_. PL is a highly sensitive technique used to determine the photocatalytic performance and reveal charge separation/recombination rates of photo-excited carriers [41]. In general, the higher PL intensity corresponds to the higher recombination rates of photogenerated electron-hole pairs and the lower photocatalytic activity. The biochar possesses the strong electron transport capacity, as indicated by the lowest PL intensity in the prepared materials. Pure g-C_3_N_4_ shows a strong peak at around 460 nm, and this is consistent with the absorption edge in the UV-vis diffuse reflectance spectra [42]. PL emission spectra of K-doped g-C_3_N_4_ exhibit similar signals to those of pure g-C_3_N_4_, but the intensity of the PL spectra is affected, indicating that doping K reduced the recombination of electrons and holes and enhanced the separation efficiency [43]. Besides, the PL intensity of biochar-supported K-doped g-C_3_N_4_ is further weakened, since the biochar can serve as efficient electron-transfer channels and acceptors to inhibit the recombination of photogenerated electron-hole pairs.

### 3.2. Photocatalytic Activity

**Figure 6:**
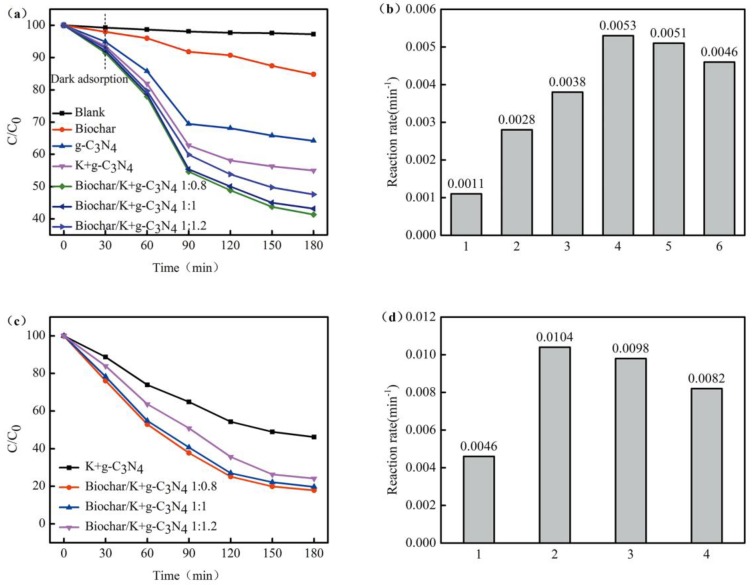
(**a**) Photocatalytic degradation curve of naphthalene in the first protocol and (**b**) the corresponding apparent rate constants (1-Biochar, 2-g-C_3_N_4_, 3-K+g-C_3_N_4_, 4-Biochar/K+g-C_3_N_4_ 1:0.8, 5- Biochar/K+g-C_3_N_4_ 1:1 and 6-Biochar/K+g-C_3_N_4_ 1:1.2). (**c**) Photocatalytic degradation curve of naphthalene in the second protocol and (**d**) the corresponding apparent rate constants (1-K+g-C_3_N_4_, 2-Biochar/K+g-C_3_N_4_ 1:0.8, 3- Biochar/K+g-C_3_N_4_ 1:1 and 4-Biochar/K+g-C_3_N_4_ 1:1.2).

The photocatalytic performances of as-prepared samples were investigated by the degradation experiments with 20-mg L^−1^ naphthalene solution under visible light illumination. Figure 6a shows the results of the first protocol. For the purpose of comparison, in the absence of photocatalysts, naphthalene degradation experiments were performed under visible light irradiation, and degradation of naphthalene was not observed, indicating that a photocatalyst was an essential factor in the photocatalytic process [44]. It is generally believed that a biochar plays an important role in the adsorption of organic pollutants, since a biochar has a large specific surface area and sufficient pore geometry [45]. Unsurprisingly, pure g-C_3_N_4_ displayed a lower degradation rate because of the high electron-hole recombination rate. For K-modified g-C_3_N_4_ showed the obviously improved naphthalene degradation rate, because doping K reduced the recombination of electrons and holes and improved the photocatalytic activity. A series of biochar-supported K-doped g-C_3_N_4_ compound materials showed the much higher activity than K-modified g-C_3_N_4_, since the biochar acted as a cocatalyst to provide abundant catalytic sites. Moreover, the biochar had a strong affinity to organic pollutants, and naphthalene dissolved in the reaction system could be enriched at the surface of the biochar. Particularly, biochar-supported K-doped g-C_3_N_4_ compound materials prepared with two mass ratios (1:0.8 and 1:1) showed much higher activity than those prepared with other mass ratios, because excessive K-doped g-C_3_N_4_ powder was aggregated or crystallized on the surface of the biochar and blocked some active sites.

Figure 6c shows the degradation performances of naphthalene under the second protocol. K-doped g-C_3_N_4_ and different proportions of biochar-supported K-doped g-C_3_N_4_ showed better results under direct light exposure experiments. The results might be interpreted as follows. After the adsorption in the suspension for 30 min in the dark, active sites of the catalyst were occupied by naphthalene, thus restricting the exposure of the photocatalyst to the light source. However, when the suspension was directly illuminated, adsorption and photodegradation were carried out simultaneously. Naphthalene was degraded once it was adsorbed, thus effectively increasing the degradation rate of pollutants. The result was consistent with the previous report [46]. 

In addition, the photodegradation process of naphthalene with the photocatalysts appeared to follow the pseudo-first-order kinetics reaction. For the first protocol, the relative rate constants were calculated to be 0.0011, 0.0028, 0.0038, 0.0053, 0.0051 and 0.0046 min^−1^ for the biochar, g-C_3_N_4_, K-doped g-C_3_N_4_, biochar-supported K-doped g-C_3_N_4_ (1:0.8), biochar-supported K-doped g-C_3_N_4_ (1:1) and biochar-supported K-doped g-C_3_N_4_ (1:1.2), respectively (Figure 6b). For the second protocol, the apparent rate constants of K-doped g-C_3_N_4_, biochar-supported K-doped g-C_3_N_4_ (1:0.8), biochar-supported K-doped g-C_3_N_4_ (1:1) and biochar-supported K-doped g-C_3_N_4_ (1:1.2) samples were 0.0046, 0.0104, 0.0098 and 0.0082 min^−1^, respectively (Figure 6d).When the suspension is directly exposed to light, the relative rate constants of all K-doped g-C_3_N_4_ and biochar-supported K-doped g-C_3_N_4_ photocatalysts were higher than those of the first condition. It was observed that the biochar-supported K-doped g-C_3_N_4_ (1:0.8) performs best under visible light. The rate constant of optimal photocatalysts under second condition is about two times higher than that of the first condition.

### 3.3. Photostability

**Figure 7:**
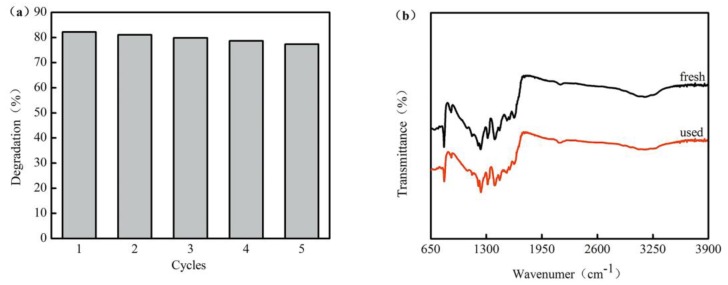
(**a**) Repetitive photocatalytic degradation of naphthalene over biochar-supported K-doped g-C_3_N_4_ (mass ratio, 1:0.8) under visible light and (**b**) FTIR spectra of biochar-supported K-doped g-C_3_N_4_ (mass ratio, 1:0.8) before and after the photocatalytic reaction.

The stability and reusability of the prepared photocatalysts largely determine their application. In order to investigate the stability of the catalyst, the photocatalytic degradation of recycled biochar-supported K-doped g-C_3_N_4_ (1:0.8) was carried out according to the second protocol. The photocatalytic activity of the recovered photocatalyst showed no noticeable decrease after five consecutive cycles (Figure 7). The comparison of the FTIR spectra of the photocatalysts before and after the reaction indicated that the chemical structure was not changed. Therefore, it was confirmed that the biochar-supported K-doped g-C_3_N_4_ photocatalyst was not photo-corroded and had high stability during the photocatalytic degradation process. The high stability might be ascribed to the biochar, which acted as a cocatalyst and was well-connected with carbon nitride.

### 3.4. Photocatalytic Degradation Mechanism of Naphthalene

#### 3.4.1. Trapping Experiments and Formation of Active Species

**Figure 8:**
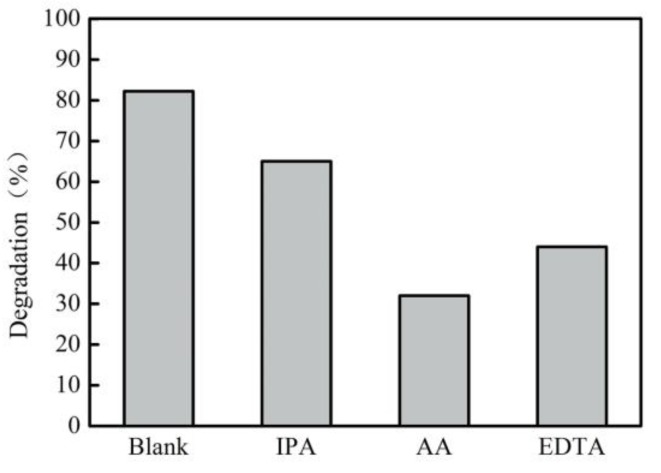
Photodegradation rates of naphthalene in the presence of different scavengers (IPA-isopropanol, AA-ascorbic acid, EDTA-ethylenediamine tetraacetic acid).

In order to further elucidate the active species of naphthalene degradation in the photocatalytic reaction system, trapping experiments were conducted. In this work, isopropanol (IPA), ascorbic acid (AA) and ethylenediamine tetraacetic acid (EDTA) were employed as the hydroxyl radical (▪OH), superoxide radical anion (▪O_2_^−^) and hole (h^+^) quencher, respectively [46]. The results are shown in Figure 8. When the IPA, the typical scavenger of ▪OH, was used, the photodegradation rate of naphthalene reached 65%, indicating that ▪OH played an important role in the photodegradation of naphthalene. Moreover, when AA and EDTA were respectively added, the degradation rates were respectively 32% and 44%, indicating that ▪O_2_^−^ and h^+^ were also the dominant active species.

**Figure 9:**
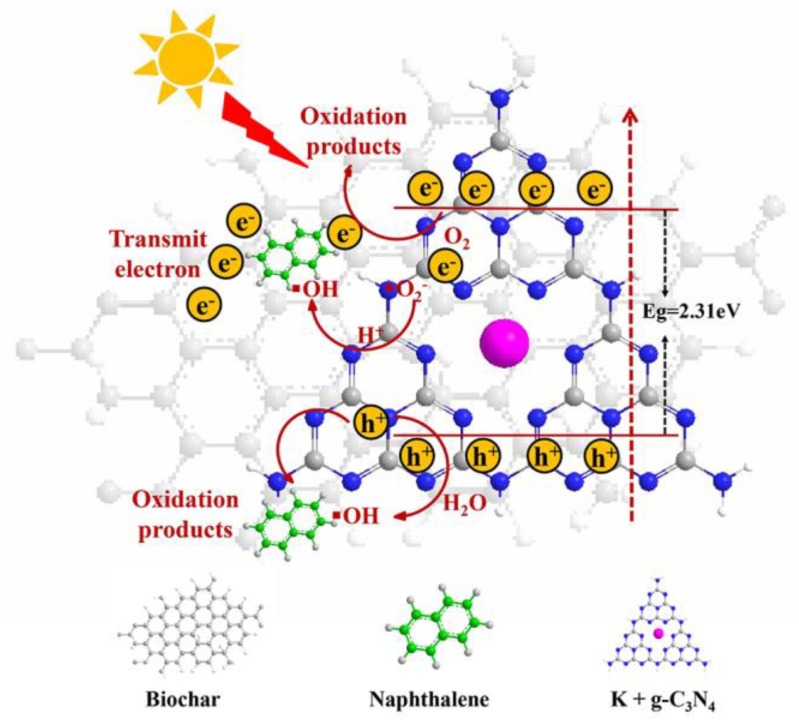
Schematic diagram of a photogenerated charge transfer in the biochar-supported K-doped g-C_3_N_4_ system under visible light irradiation.

Under visible light irradiation, g-C_3_N_4_ is activated to move electrons from the valence band (VB) to the conduction band (CB), followed by the formation of electron hole pairs [47]. The electrons on CB rapidly transfer to the biochar, because the biochar has the good electronic transmission ability. Then, a part of the electrons on the CB by the biochar transfer directly and serve as the terminal electron acceptor. The lifetime of photogenerated electrons is prolonged, thus effectively promoting charge separation and facilitating the photocatalytic reaction. Photogenerated electrons transferred to the biochar surface can react with O_2_ dissolved in the reaction solution to form superoxide radical anion (▪O_2_^−^). ▪O_2_^−^ can also generate ▪OH through a series of chemical reactions [48]. Meanwhile, photogenerated holes can react with H_2_O to form hydroxyl radicals (▪OH). Both ▪O_2_^−^ and ▪OH can oxidize naphthalene. In addition, holes (h^+^) can also be used as an active species to undergo the oxidation reaction with naphthalene, ultimately producing CO_2_, H_2_O and other small molecules (Figure 9) [49].

#### 3.4.2. Intermediates of Naphthalene Degradation

The intermediates of the photocatalytic degradation of naphthalene were determined by the GC-MS technique. The main intermediate products are listed in Table 1.

#### 3.4.3. Reaction Pathway

**Figure 10:**
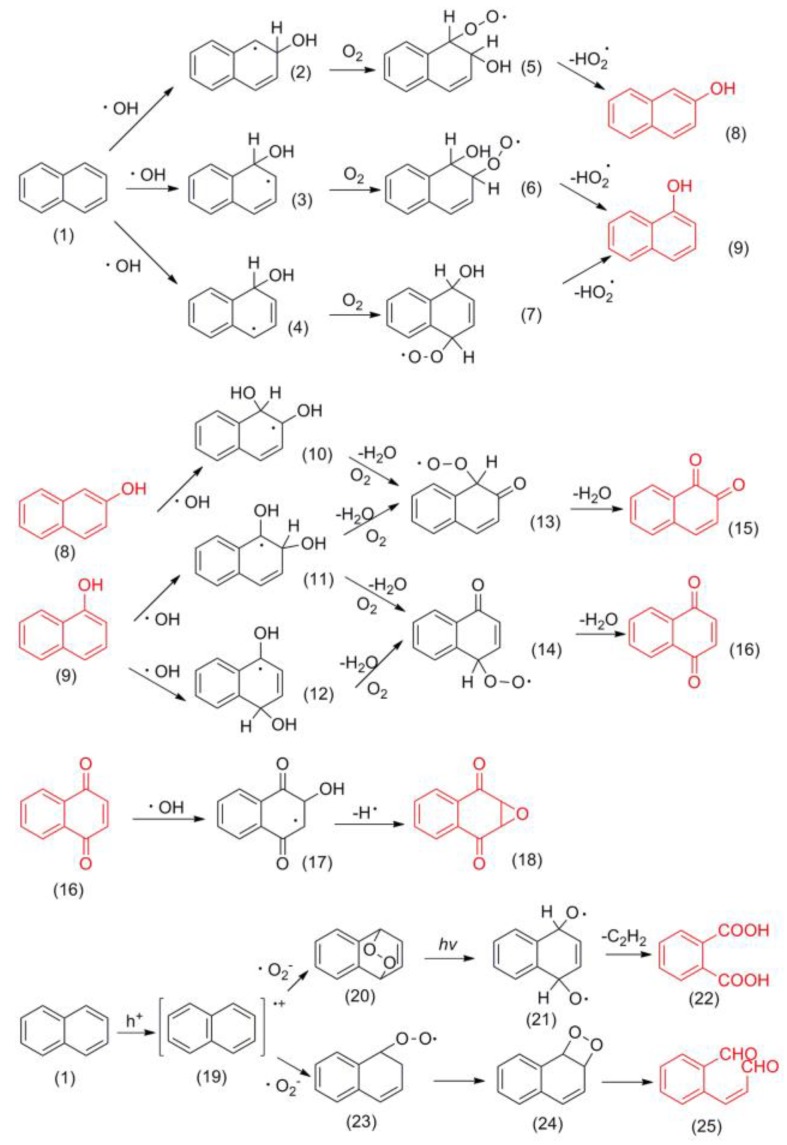
Possible pathways for the formation of the main intermediates of naphthalene degradation.

Based on the detection results of the main intermediates, the reaction pathway of naphthalene degradation is proposed (Figure 10). Firstly, naphthalene (1) is attacked by ▪OH either at the α position or the β position to generate three different hydroxylated naphthalene radicals (2,3 and 4), which can react with O_2_ and subsequently eliminate HO_2_▪ to generate 1-naphthol (8) and 2-naphthol (9) [50]. Secondly, 1-naphthol and 2-naphthol react with another ▪OH to form the corresponding dihydroxyl radicals (10,11 and 12), which can eliminate H_2_O and react with O_2_ to generate carbonyl peroxyl radicals (13 and 14). Thirdly, the carbonyl peroxyl radicals (13 and 14) capture a hydrogen atom from the solvent and eliminate H_2_O to form 1,2-naphthalenedione (15) and 1,4-naphthalenedione (16) [51]. Finally, the double-bond on the nonaromatic ring of 1,4-naphthalenedione reacts with ▪OH, then eliminates the hydrogen radical and eventually produces 2,3-dihydro-2,3-epoxy-1,4-naphthoquinone (18) [52].

In addition to ▪OH, h^+^ and ▪O_2_^−^ are also important active species. Naphthalene (1) and a photogenerated hole undergo an electron transfer reaction to generate a naphthalene radical cation (19), which can react with the superoxide anion to form the endoperoxide (20). The endoperoxide (20) continues to decay into a diradicalic species (21) under illumination, which forms 1,2-dicarboxybenzene (22) through an elimination reaction and oxidation reaction. Naphthalene radical cation (19) can also react with a superoxide anion to form a peroxide species (23), which is then dissociated into 2-formylcinnamaldehyde (25). The main intermediates and the proposed mechanism were in agreement with previous studies [50].

## 4. Conclusions

In summary, the novel visible-light-driven biochar-supported K-doped g-C_3_N_4_ photocatalyst was synthesized by a facile calcination-impregnation method. The properties of the biochar-supported K-doped g-C_3_N_4_ photocatalyst, such as optical and catalytic properties and absorption capacity, were improved by the introduction of potassium and biochar. The biochar-supported K-doped g-C_3_N_4_ composites with the mass ratio of 1:0.8 exhibited the highest naphthalene degradation efficiency of 82.19% within 180 min under visible light irradiation due to the synergetic effects between K-doped g-C_3_N_4_ and biochar. The introduction of potassium effectively inhibited the growth of g-C_3_N_4_ grains, reduced the band gap energy and enlarged the response range to visible light. In addition, the biochar, as electron transfer channels and acceptors, effectively inhibited the recombination of photogenerated electron-hole pairs and had a large number of surface hydrophilic functional groups, which could effectively improve the dispersion of the prepared composite photocatalytic materials in polar solvents such as water and further improve the photocatalytic performance. Meanwhile, the biochar-supported K-doped g-C_3_N_4_ photocatalyst possessed good stability without a significant activity loss after five degradation cycles. Hence, the biochar-supported K-doped g-C_3_N_4_ photocatalyst could be considered a promising material for the degradation of organic pollutants.

## Figures and Tables

**Table 1:** Main reaction intermediates identified by gas chromatography-mass spectrometer (GC-MS).

Nos.	Structures	Names	Nos.	Structures	Names
1	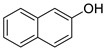	2-naphthol	2	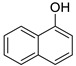	1-naphthol
3	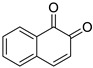	1,2-naphthalenedione	4	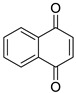	1,4-naphthalenedione
5	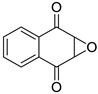	2,3-dihydro-2,3-epoxy-1,4-naphthoquinone	6	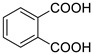	1,2-dicarboxybenzene
7	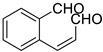	2-formylcinnamaldehyde			
